# Unraveling past impacts of climate change and land management on historic peatland development using proxy‐based reconstruction, monitoring data and process modeling

**DOI:** 10.1111/gcb.14298

**Published:** 2018-05-30

**Authors:** Andreas Heinemeyer, Graeme T. Swindles

**Affiliations:** ^1^ Environment Department Stockholm Environment Institute University of York York UK; ^2^ water@leeds School of Geography University of Leeds Leeds UK

**Keywords:** carbon budgets, carbon emissions, MILLENNIA model, peat accumulation, peatland management, peatlands, testate amoebae, water‐table

## Abstract

Peatlands represent globally significant soil carbon stores that have been accumulating for millennia under water‐logged conditions. However, deepening water‐table depths (WTD) from climate change or human‐induced drainage could stimulate decomposition resulting in peatlands turning from carbon sinks to carbon sources. Contemporary WTD ranges of testate amoebae (TA) are commonly used to predict past WTD in peatlands using quantitative transfer function models. Here we present, for the first time, a study comparing TA‐based WTD reconstructions to instrumentally monitored WTD and hydrological model predictions using the MILLENNIA peatland model to examine past peatland responses to climate change and land management. Although there was very good agreement between monitored and modeled WTD, TA‐reconstructed water table was consistently deeper. Predictions from a larger European TA transfer function data set were wetter, but the overall directional fit to observed WTD was better for a TA transfer function based on data from northern England. We applied a regression‐based offset correction to the reconstructed WTD for the validation period (1931–2010). We then predicted WTD using available climate records as MILLENNIA model input and compared the offset‐corrected TA reconstruction to MILLENNIA WTD predictions over an extended period (1750–1931) with available climate reconstructions. Although the comparison revealed striking similarities in predicted overall WTD patterns, particularly for a recent drier period (1965–1995), there were clear periods when TA‐based WTD predictions underestimated (i.e. drier during 1830–1930) and overestimated (i.e. wetter during 1760–1830) past WTD compared to MILLENNIA model predictions. Importantly, simulated grouse moor management scenarios may explain the drier TA WTD predictions, resulting in considerable model predicted carbon losses and reduced methane emissions, mainly due to drainage. This study demonstrates the value of a site‐specific and combined data‐model validation step toward using TA‐derived moisture conditions to understand past climate‐driven peatland development and carbon budgets alongside modeling likely management impacts.

## Graphical abstract

We compared testate amoebae (TA)‐based water table (WTD) reconstructions to instrumentally monitored WTD and hydrological model predictions using the MILLENNIA peatland model to examine past peatland responses to climate change and potential grouse moor land management including drainage and rotational vegetation burning. The comparison of TA vs. MILLENNIA model and measured data revealed striking similarities in overall WTD patterns. However, there were clear periods when TA‐based predictions underestimated WTD. Importantly, simulated grouse moor management scenarios seemed to explain this period of drier TA WTD predictions, resulting in considerable model predicted carbon losses and reduced methane emissions, mainly due to drainage.

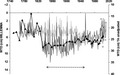

## 1 INTRODUCTION

Globally, peatlands contain ~30% of all soil organic carbon (SOC), despite covering only 3% of the land surface (Parish et al., 2008). They occur mainly in the Northern Hemisphere circumpolar region, where low temperatures, high soil moisture and slow decay rates of litter input via net primary production (NPP) allow peat to form (i.e. a long‐term positive balance between NPP and litter decay), often under conditions of high water‐table depth (WTD). Crucially, this slow decay preserves an archive of peatland development (i.e. animal and plant remains) that can be dated and used to reconstruct past drivers of peatland growth, such as WTD and vegetation composition, providing key information on how peatlands respond to changes in climate.

Blanket bogs are a globally rare peatland habitat with the United Kingdom containing about 15% of this habitat (Tallis, 1998) but mostly in a degraded state (Natural England, 2008), largely due to past environment (e.g. N deposition) and management (e.g. drainage) impacts. In the UK blanket bogs represent about 90% of all peatlands (Bain et al., 2011), which are often managed for grazing and grouse shooting, commonly supported by draining the peat and regular burning of vegetation. The consequence is dominance of heather (*Calluna vulgaris*), very low overall plant biodiversity, suppressed cover of peat‐forming *Sphagnum* mosses (Lindsay, 2010), and often drier and eroding peat. In fact, only about 12% of protected blanket bog sites are classified as in favorable condition (Natural England, 2008). However, little is known about management impacts on the long‐term SOC accumulation and soil C emissions.

UK blanket bogs have accumulated peat since the start of the Holocene about 11.6 k years ago under varying climate, but current bioclimatic models highlight the threat by climate change to their natural range (Gallego‐Sala et al., 2010), suggesting that they might start to degrade as the climate warms (Gallego‐Sala & Prentice, 2013) resulting in water‐table drawdown and increased decomposition. However, existing bioclimatic models do not consider extremely important autogenic negative feedbacks within peatlands that may actually act as a “buffer” to climate change (Swindles, Morris, Baird, Blaauw, & Plunkett, 2012). Such feedbacks include ecohydrological linkages between changes in WTD and shifts in vegetation communities (with different rooting depth and litter quality and thus affecting SOC inputs across depth and peat decomposition rates). A better process‐level understanding of climate‐peatland SOC feedbacks is clearly needed (Davidson & Janssens, 2006) since the mineralization of peatland soil organic matter (SOM) has the potential to release vast amounts of previously locked‐up C into the atmosphere. Depending on the water‐table level, C emissions from decomposition are either as CO_2_ (oxic acrotelm) or CH_4_ (anoxic catotelm), the latter particularly responsible for exacerbating climate change and affecting the overall greenhouse gas (GHG) emissions. However, a key limiting issue in more accurate predictions of future climate is still the inadequate model representation of climate – terrestrial carbon (C) cycle feedbacks, particularly of peatland soil organic carbon (Limpens et al., 2008). Several peatland models of varying complexity and feedback mechanisms have been developed (Baird, Morris, & Belyea, 2012; Bauer, Gignac, & Vitt, 2003; Clymo, 1984; Frolking et al., 2010; Gignac, Vitt, & Bayley, 1991; Heinemeyer et al., 2010), which have often been compared to measured C stocks. However, there is a lack of validating the C cycle underpinning hydrological model predictions against past palaeo records.

Peat archives from peat cores are also important for testing peatland development models, which predict peat layer accumulation and their associated chemical (e.g. carbon content) and physical (e.g. bulk density) properties, enabling comparison between the two. Data‐model comparisons have revealed uncertainties in peat accumulation processes (Clark et al., 2010), particularly considering hydrology, vegetation and NPP. Furthermore, recent peat core studies (Charman et al., 2013) indicate increased C accumulation during warmer periods due to increased NPP outweighing higher decomposition, which contradicts most global earth system model carbon cycle simulations (Friedlingstein et al., 2006). Moreover, those global earth system models used by the Intergovernmental Panel on Climate Change (IPCC) do not yet adequately include peatland SOC dynamics, limiting global predictions on future climate C‐cycle feedbacks and resulting GHG emissions. Latest attempts to include peatlands in global dynamic vegetation models (e.g. LPX; Spahni, Joos, Stocker, Steinacher, & Yu, 2013) are promising, yet still show major process‐level uncertainties.

Testate amoebae (TA) are commonly used to reconstruct peatland hydrological changes over the Holocene, as indicator species are aligned across a gradient of wet to dry conditions based on present‐day associations (Amesbury et al., 2016; Turner, Swindles, & Roucoux, 2014). Peat cores provide a stratigraphic (i.e. temporal) archive of past TA species composition, thus allowing to predict past moisture (and likely water‐table) conditions from understanding of contemporary ecology. Quantitative transfer functions are used to establish a relationship between present‐day species composition and hydrological data and then applied to subfossil data from cores (Amesbury et al., 2016).

Model predictions of peat carbon stock and flux changes rely on capturing seasonal and interannual WTD changes. Although in the short‐term site measurements can be used for model validations, validation over longer‐time scales relies on comparing model predictions to WTD reconstructions, for example, based on TA taxa found within the peat core. In addition to other dating tools such as radiocarbon (Turner et al., 2014), the use of Spheroidal Carbonaceous Particles (SCPs; Swindles, 2010) allows the generation of temporally constrained records of palaeo‐hydrological conditions in the recent past. Together with dating tools, palaeo‐reconstructions thus offer crucial insights into peatland development, (i) understanding baseline trajectories in peatland development, and (ii) assessing long‐term resilience and recovery rates of peatlands to climate or management impacts (Swindles et al., 2016). TA based reconstructions of past WTDs can then be compared to process model predictions, providing an important hydrological model validation tool. A good fit between TA and model predicted WTDs supports applying model scenarios to explore past management impacts on peatland functioning and C storage. The MILLENNIA peatland model predicts peat hydrological conditions (i.e. water‐table depth) based on climatic conditions and long‐term peat column growth either for annual or monthly time steps. Here, we used the annual MILLENNIA version (Heinemeyer et al., 2010) for long‐term peat accumulation during the Holocene until 1914, and then either the annual or monthly version (Carroll et al., 2015) until 2012. This choice of model application reflected the availability of climate data. The WTDs predicted by the MILLENNIA model allowed comparison to TA‐based WTD reconstructions from ca. 1760 onwards for a peat core at Moor House National Nature Reserve (NNR) using TA transfer functions. The WTDs from different management model scenarios were then compared to the TA‐based WTD, and related to C accumulation and C emissions affecting the GHG balance.

## 2 MATERIALS AND METHODS

### 2.1 Site location and environmental conditions

The study site, Moor House National Nature Reserve (NNR), covers about 75 km^2^ of a typical UK blanket bog with much known about its ecology (Garnett, Ineson, Stevenson, & Howard, 2001; Heal & Perkins, 1978) together with detailed meteorological (i.e. weather station) and hydrological (i.e. water‐table) data collected by the Environmental Change Network (ECN). It is located in the northern Pennines across an elevation range of 290–850 m (a.s.l.) and is characterized by a subarctic–oceanic climate with an average long‐term mean annual temperature (MAT) and precipitation (MAP) at 550 m altitude of about 5.1°C (1931–1997) and around 2,000 mm (last 20 years), respectively (cf. Garnett, 1998). Peat formation at the study site started about 9,000 years ago (see Heinemeyer et al., 2010 for more site information). This study focuses on a square kilometre around the ECN meteorological station at 550 m (NY757328; 54°68′ N, 2°37′W), a site with mostly deep (>1 m) peat (histosol), supporting dominant vegetation of *Calluna vulgaris* L. [Hull] and *Eriophorum* spp. with some *Sphagnum* spp., and classified as a *Calluna vulgaris–Eriophorum* blanket mire (for more information on vegetation and peat depth see Garnett, Ineson, & Stevenson, 2000 and Heinemeyer et al., 2010). Typically the peatland site has a pH of generally between 3.0 and 4.2 and a high mean annual WTD of within 5 cm of the peat surface for >80% during the year (Evans, Burt, Holden, & Adamson, 1999).

### 2.2 Peatland model predictions

The MILLENNIA peatland model (Heinemeyer et al., 2010) predicts peat hydrological conditions (i.e. water‐table) based on climatic conditions either for annual or monthly time steps. The hydrological conditions, in connection with internal factors (e.g. litter quality) and external modifiers (e.g. temperature and oxygen availability), then determine decomposition rates of old and new carbon fractions (as in Bauer, 2004) across the peat profile based on litter inputs via NPP as a function of potential evapotranspiration (PET) based on Lieth and Box (1972). Detailed model explanations are provided in Heinemeyer et al. (2010; annual model) and Carroll et al. (2015; monthly model). Here, we used the annual version for the long‐term peat accumulation during the Holocene and for the period until 1914, and either the annual or monthly version until 2012 (reflecting model application and climate data availability). However, model outputs are only provided as annual averages in relation to average TA predictions. The main model driver is climate with either monthly or annual temperature and rainfall amounts as inputs. Both versions consider topography (e.g. slope affecting temperature, runoff and erosion), vegetation (e.g. affecting NPP, litter quality and transpiration losses) and proceeding hydrological conditions (e.g. high water‐tables leading to higher run‐off) to predict a new water‐table, vegetation (based on the preceding 5‐year average water‐table) and corresponding changes in soil carbon fluxes (i.e. CO_2_ and CH_4_ from decomposition), peat depth increments (i.e. accumulation) and soil C budgets (change in soil C stock in relation to input from NPP and losses from soil C fluxes from decomposition and erosion).

Further changes were implemented to improve hydrological and C flux process representation by calculating water filled pore space in the peat, bedrock drainage, plant‐mediated transport and methane oxidation (oxidation):

- *The available pore space* in relation to the height above the water‐table depth (WTD) was based on data by Hayward and Clymo (1982; see Figure 4, but ignoring the short term hysteresis effect); an exponential relationship is assumed between the distance to the water‐table and the available pore space (0.2*EXP(1.6*WTD^2^)), such that available space increases with distance from the water‐table. Total space is then calculated by integrating the available pore space over the available unsaturated peat cohorts. By combining the water entering the system with the available space, a new WTD is calculated.
- *To simulate drainage* of the peat column into the bedrock, two drainage factors are included, specific yield (SY) and hydraulic conductivity (HC). These are set to default values of 0.02 (SY in %) and 0.1 (HC in cm/year) reflecting average values for clay reported by Johnson (1967) for SY (2%) and for unweathered clay based on Bear (1972) for HC (10–5 feet/day). However, SY and HC can be altered as a user input.
- *The plant functional type* (vegetation) composition of shrub, sedge, rush, grass, herb, *Sphagnum*, other moss) is based on a moving average of 5 years of previous water‐tables, allowing representing a more stable/resilient vegetation in the case of only a few very dry or wet years.
- *The anoxic ratio* is set to 0.035, similar to values reported in previous literature (Bauer, 2004) ranging from 0.025 to 0.0625.
- *Methane oxidation* is set to 0.05 g C g^−1^ year^−1^ and reflects the range of the very scarce data available on methane oxidation in relation to dry peat and/or on a carbon (mass) basis, i.e. McDonald, Hall, Pickup, and Murrell (1996) provided incubation values at 20°C for UK peat of around 0.08 g C g^−1^ year^−1^, Watson, Stephen, Nedwell, and Arah (1997) quoting 0.012 g C g^−1^ year^−1^ (i.e. 0.001021 mol C g^−1^ year^−1^ equal to 1.021e^−3^ mol C g^−1^ year^−1^), but Yrjälä et al. (2011) quoted only 0.0009 g C g^−1^ year^−1^ (0.2 μmol g^−1^ DW day^−1^) and Whalen and Reeburgh (2000) measured around 0.002 g C g^−1^ year^−1^.

We used available reconstructed Holocene climate data (based on a combination of recent instrumental data and a variety of existing multiproxy climate reconstructions, see Morris, Baird, Young, & Swindles, 2015) to model long‐term peat accumulation, Met Office 5 km gridded data (Perry & Hollis, 2015) for the recent past (1914–1991) and ECN data (ECN Data Centre: http://data.ecn.ac.uk) for recent present periods (1992–2012). Met Office data were adjusted for elevation for the Moor House site in order to achieve the same long‐term average temperature and rainfall amounts as the Moor House ECN data (see Carroll et al., 2015). Model predictions of monthly WTD could be compared to averaged ECN hourly automated dipwell data (UK grid location: NY 75072 33425; missing data were gap‐filled by interpolation of manual data) for the period of 1999–2012. Water‐table depth standard deviation for a Moor House model evaluation (see supplementary data in Carroll et al., 2015) was predicted to within 0.32 cm (linear *R* ^2^ = 0.57).

Moor House was a formal shooting estate during 1842–1951 and grouse moor management scenarios reflected available site information (Rob Rose, CEH; personal communication), which indicated a 20 year burn rotation. The associated drainage was assumed to last from 1831, before intensification of grouse shooting (to enable enhanced heather growth and drier access conditions for gamekeepers), until the late 1950s. Burning was anticipated to have started in 1851 and to reduce NPP to 1% in the burnt year (and charcoal adding about 5% to an inert carbon pool), subsequently recovering in a sigmoidal shape to 100% by either 5 or 10 years after burning (based on field observations by A. Heinemeyer et al., *unpublished data*). Drainage was expected to reduce WTD on average by 5 cm, based on the field evidence of Wilson et al. (2010). Reduced WTD were linked to enhanced decomposition and increased the associated CO_2_ but decreased CH_4_ emissions similarly to modeled impacts of natural WTD changes (Heinemeyer et al., 2010). Drainage (grip) effectiveness was assumed to be at optimum for 25 years and declining to 60% over the subsequent 15 years (renewed once in 1871 and then maintained at optimum until 1905, reflecting intense grouse shooting), finally declining to 0% by 1955. Grazing pressure was anticipated to be insignificant above 450 m (i.e. no reduction in NPP at the modeled altitude of 550 m a.s.l.). Further model scenarios considered a no management option (no shoot).

### 2.3 Water‐table reconstructions

A Russian peat core was taken from the top 50 cm of peat beside the Moor House ECN station (Lat. 54.695500; Lon. −2.387400) following De Vleeschouwer, Chambers, and Swindles (2010). The core was returned to the laboratory and stored at 4°C before analysis. The top 20 cm of the core was sampled at 0.5 cm resolution and TA were prepared using the standard method of Booth, Lamentowicz, and Charman (2010). We applied the Northern England and pan‐European transfer functions to the subfossil TA data to reconstruct WTD (Amesbury et al., 2016; Turner, Swindles, Charman, & Blundell, 2013). In both cases weighted‐averaging tolerance downweighted regression with inverse deshrinking was used as it yielded the best performance statistics. The water‐table reconstructions were standardized following Swindles et al. (2015). There is some variation in predicted water‐tables between the two transfer functions which is caused by intermodel differences in WTD optima of key taxa. Some drier taxa (e.g., *Nebela militaris*,* Trigonopyxis arcula* type) have drier optima in the Northern England model owing to the inclusion of some very dry peatlands affected by fire and drainage from this region. Sample‐specific standard errors for the TA transfer function reconstructions were based on 1,000 bootstrapping cycles (Amesbury et al., 2016). The resulting WTD predictions could then be paired with mean annual WTD predictions for years obtained from the MILLENNIA model based on establishing a peat age‐depth profile based on a SCP profile.

### 2.4 Peat core age‐depth profile

SCPs have been deemed to provide reliable age information from peatlands in Northern Britain and Ireland for the last ~150 years (Swindles, 2010; Swindles, Blundell, Roe, & Hall, 2010; Turner et al., 2014). Three unambiguous features can be used as age‐equivalent stratigraphic markers: (i) the start of the record (c. 1850), (ii) the rapid increase in SCPs (c. 1950) and (iii) the peak (c. 1978). These represent (i) the start of high temperature combustion and power generation; (ii) the post‐WW2 increase in power generation and fossil fuel burning and (iii) the peak of SCP production before reliance on other methods of power generation and the advent of clean fuel technologies (Rose, Harlock, Appleby, & Battarbee, 1995). We used the method of Swindles (2010) to prepare SCPs from the peat. Linear interpolation was used to generate a simple age‐depth model between the three SCP age‐equivalent stratigraphic markers and the date of core sampling (2011) as the uppermost time point; dates before the start of the SCPs are an extrapolation based on accumulation rate.

## 3 RESULTS

### 3.1 Water table reconstructions

**Figure 1:**
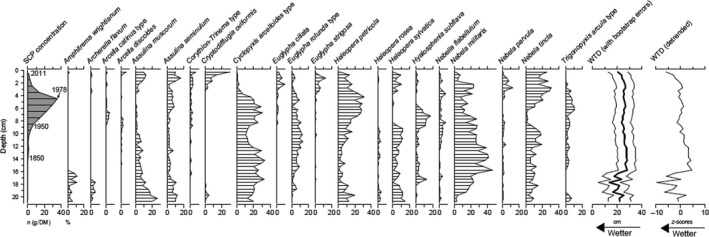
Testate amoebae record (%) from the Moor House blanket bog, Northern Britain alongside the SCP concentration data with chronological assignments marked (i.e. 1978 at ~4.5 cm; 1950 at ~9.0 cm; 1850 at ~14.0 cm). The water‐table depth (WTD) reconstruction using the transfer function of Turner et al. (2014) is illustrated with standard errors derived from bootstrapping (1,000 cycles). The standardized water‐table reconstruction in relation to *z*‐scores (following Swindles et al., 2015) is also shown

The testate amoebae data from Moor House (Figure 1) along with the SCP concentrations and water‐table reconstructions show periods of changes in wetness across the peat depth profile.

**Figure 2:**
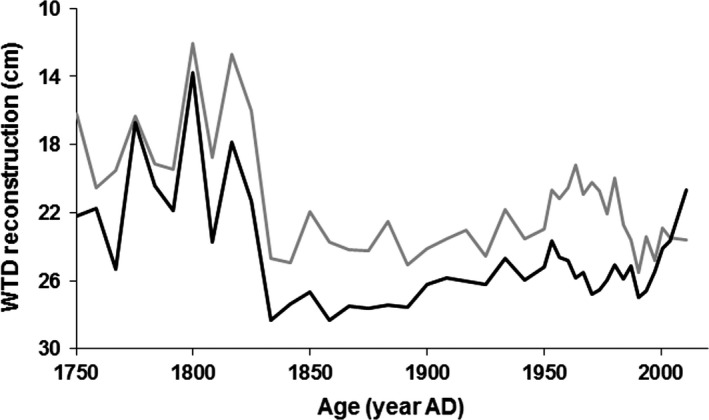
Water‐table depth (WTD) reconstructions for the Moor House blanket bog peat core based on alternative testate amoebae transfer functions: Europe, EU (gray) (Amesbury et al., 2016) vs. Northern England, NE (black) (Turner et al., 2013). Note, a lower WTD value means wetter conditions. The linear regression for the reconstructed WTD comparison is *y*_[_ _NE__]_ = 0.79*x*_[_ _EU__]_ + 7.58; *r* ^2^ = 0.65

The earliest part of the record (before 1850) is very wet owing to the presence of wet‐indicator species such as *Amphitrema wrightianum* and *Archerella flavum*. After this, the assemblage suggests quite a dry peatland surface with the abundance of *Nebela militaris*,* Cyclopyxis arcelloides* type (mostly comprised of *Phryganella acropodia*) and *Heleopera petricola*. All TA taxa identified in fossil samples were present in the modern training sets of the two transfer functions. The comparison of the Northern England and pan‐European modeled water‐tables (Figure 2) illustrates that the WTD optima of TA species from the wider European area are wetter than those for Northern England (for the regression equation see Figure 2 legend).

### 3.2 TA‐derived vs. MILLENNIA model water‐table predictions and site records

**Figure 3:**
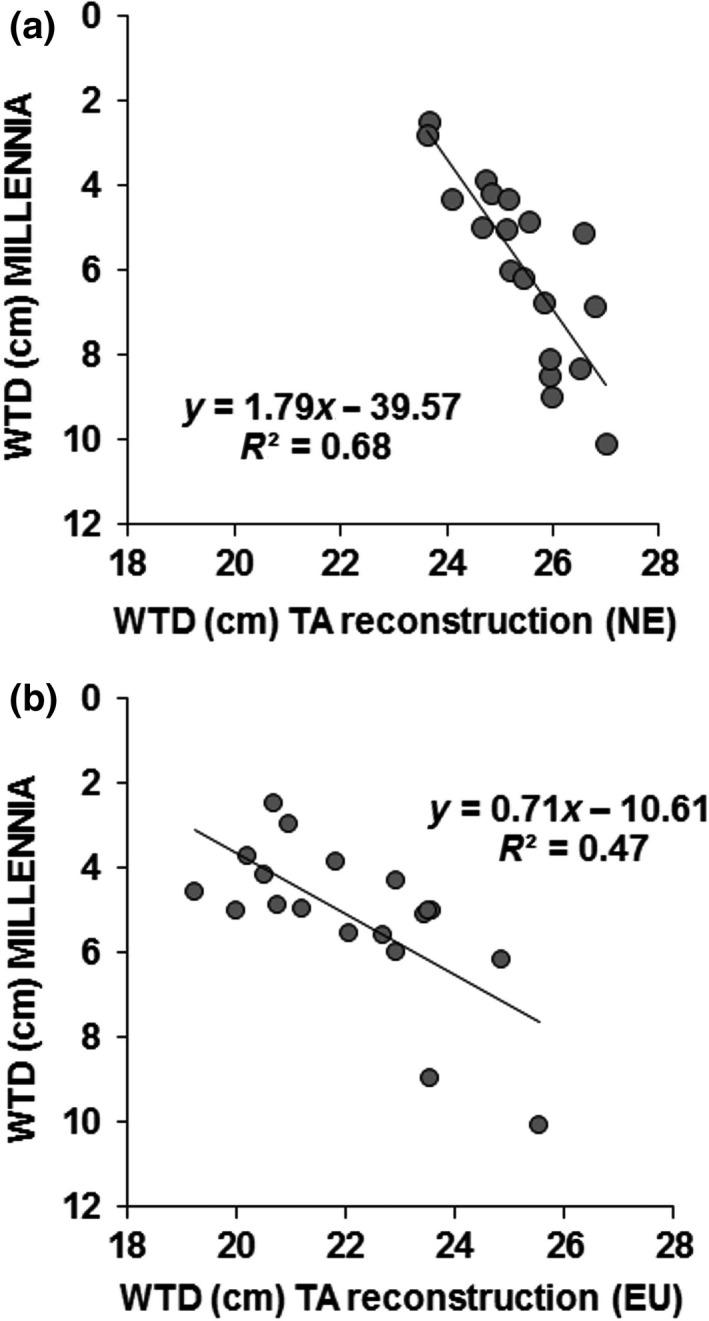
Linear regression of MILLENNIA model predicted mean annual water‐table depth (WTD) for 19 years during 1933–2004 vs. the corresponding years for the two testate amoebae (TA) reconstructions (see Figure 2) based on the (a) uncorrected transfer function derived for northern England (NE) as in Turner et al. (2013), and (b) the uncorrected European (EU) transfer function (Amesbury et al., 2016). The corresponding regression equations are shown

Water‐table reconstructions for the Moor House core based on the Northern England transfer function (WTD_TA_NE_) showed an overall good correlation with the MILLENNIA modeled WTD during the period of high quality climate records available for modeling (i.e. 1933–2004; see Figure 3a) but TA‐predictions were consistently drier (WTD = 1.79*WTD, _TA_NE_ – 39.57; *r* ^2^ = 0.68). A comparison with the EU TA transfer function (Figure 3b) indicated slightly wetter conditions but with a less good fit to the measured data (WTD = 0.714*WTD_TA_EU_ – 10.613; *r* ^2^ = 0.47). The reason behind this difference in TA‐predicted WTD likely reflects different WTD optima of key testate amoebae species in the different geographical areas. Therefore, we decided to use the WTD_TA_NE_ model for subsequent analysis because of the better directional fit in addition to closer geographical location of the TA records.

**Figure 4:**
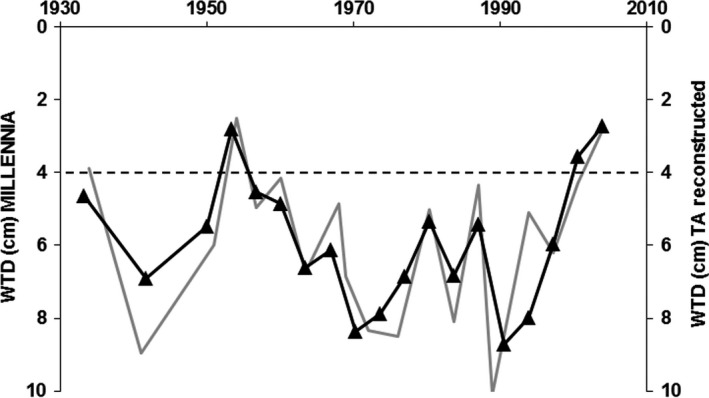
Selected years for mean annual water‐table depth (WTD) predictions (in the peat profile) between 1931 and 2004 from the MILLENNIA model (gray line) paired with years (triangles) of available testate amoebae (TA) based predictions (black line) using the offset regression (Figure 3a). The dashed line indicates the long‐term (1995–2012) mean annual WTD at Moor House (ECN data)

The available ECN site climate data (1931–2010) for Moor House were used to make comparisons between annual mean WTD predicted by the MILLENNIA model to the paired years (±1 year) with the offset corrected available TA‐derived WTD_TA_NE_ from the Moor House core (Figure 4). Not only did the WTD range predicted by the model agree with the TA‐derived WTD, but it also reproduced the general pattern of dry vs. wet years (MILLENNIA WTD = WTD_TA_NE_ + 0.004; *r* ^2^ = 0.68). Both WTD predictions indicated that the years between 1965 and 1995 were an extended period of fairly dry conditions compared to the long‐term WTD average of 4.4 ± 1.8 cm (based on annual averages of ECN dipwell data for 1995–2012).

**Figure 5:**
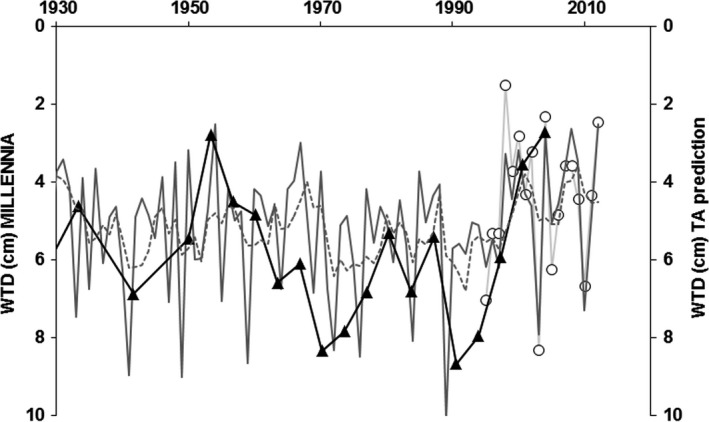
Annual water‐table depth (WTD) predictions (in the peat profile) since 1930 from the MILLENNIA model (dark gray lines, with 4‐year average as dashed line) vs. the 4‐yearly predictions (black line) from testate amoebae (TA) based on the offset regression (Figure 3a) and actual annual site WTD averages (white circles and light gray line) based on available continuous WTD data (1995–2012)

The annual MILLENNIA model prediction of WTD during 1931–2012 also showed very good agreement with the paired years of available offset corrected TA‐derived WTD, capturing peaks and troughs as well as the average trend (Figure 5). Moreover, the model predicted WTD (WTD_Mod_) followed the available annual ECN site WTD (WTD_ECN_) data very well (WTD_ECN_ = WTD_Mod_*0.813 + 0.595, *r* ^2^ = 0.57; see Carroll et al., 2015).

### 3.3 Comparison of predicted water‐tables for management scenarios and site records

**Figure 6:**
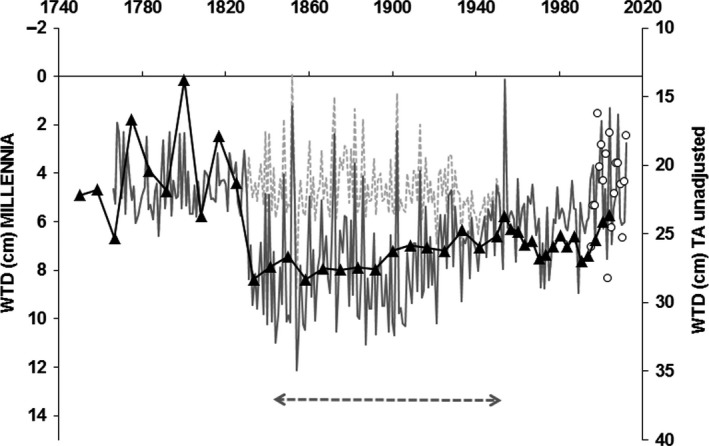
Water‐table depth (WTD) predictions from the MILLENNIA model (gray lines) vs. 4‐yearly testate amoebae (TA) based predictions (black line) and actual annual site WTD averages (white circles) based on available continuous site WTD data (1995–2012). Light dashed gray line indicates model scenario without any management, whereas dark solid gray line indicates a grouse moor management scenario during 1842–1951 (dashed arrow) with a period of active drainage (1831–1920). Note the different *y*‐axis scales (i.e. unadjusted TA WTD predictions)

Use of the available long‐term climate data for a nearby Northern England peatland site (Morris et al., 2015), adjusted to the long‐term mean temperature and total rainfall for Moor House, together with Moor House ECN climate data since 1931, provided a basis for a comparison of the WTD predicted by the MILLENNIA model over an extended time period (1750s till 2012), for which TA WTD reconstructions were available (Figure 6). Although the general WTD patterns agreed very well between the two predictions, the (unadjusted) TA WTD predictions were much drier than the MILLENNIA predictions. Whilst MILLENNIA model predictions indicated mean annual WTD conditions during 1760–1830 of between 2–6 cm, the unadjusted TA predictions of WTDs were around 15 to 25 cm. Adjusting for the 1931–2004 determined TA‐WTD offset (Figure 3a) increased the TA‐based WTD predictions to above the peat surface (−2.92 ± 6.16 cm; data not shown). However, whereas MILLENNIA model predictions of the unmanaged (no shoot) scenario remained very wet during 1830–1940, unadjusted TA‐predictions showed much lower WTD during that period, but overlapped again with the MILLENNIA model predictions from the 1940s onwards (Figure 6).

### 3.4 Predicted impacts on soil C emissions, peat C budgets and peat accumulation rates

**Figure 7:**
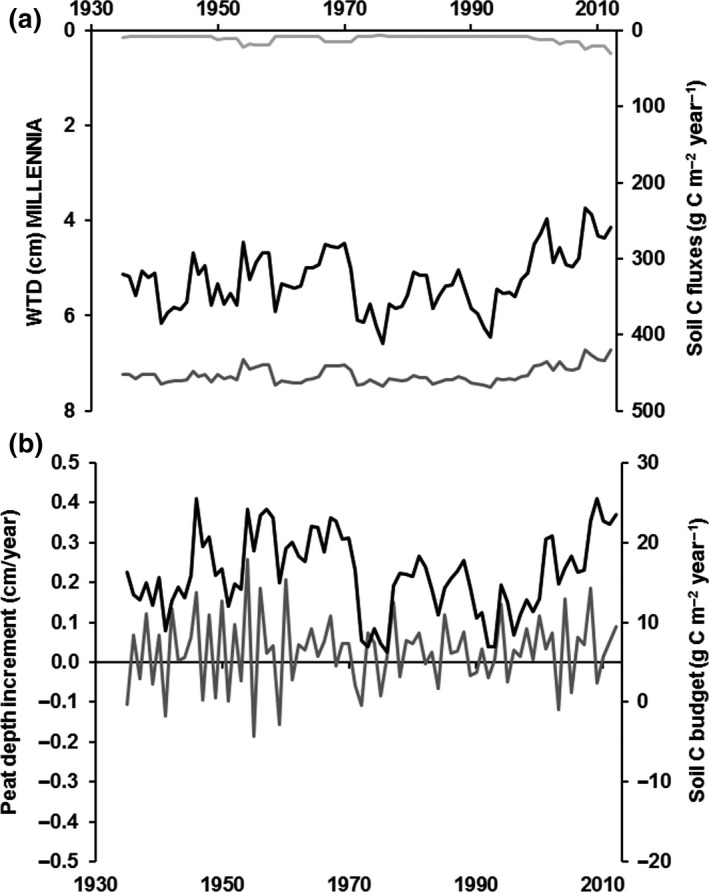
MILLENNIA model predictions (5‐year running means) of climatic impacts during 1931 to 2012 on (a) annual water‐table depth (WTD; black line) and carbon (C) fluxes of CO_2_ (dark gray line) and CH_4_ (light gray line), and (b) annual peat depth increments (gray line) and soil C budget (black line) with positive numbers representing C gains

The observed drier than average mean annual WTD predictions from the MILLENNIA model during 1965–1995 (Figure 4) corresponded to 5.2 g C m^−2^ year^−1^ lower net methane (CH_4_) but 13.4 g C m^−2^ year^−1^ higher CO_2_ emissions (Figure 7a) than during the remaining period of 1931–2012. This change in soil C fluxes to the atmosphere reflected the increased aerobic decomposition processes under a lower WTD, which resulted in near zero mean peat depth increment (reduced by 0.041 cm/year) and a lower mean soil C budget (reduced by 6.2 g C m^−2^ year^−1^) (Figure 7b) during the drier 1965–1995 period.

**Figure 8:**
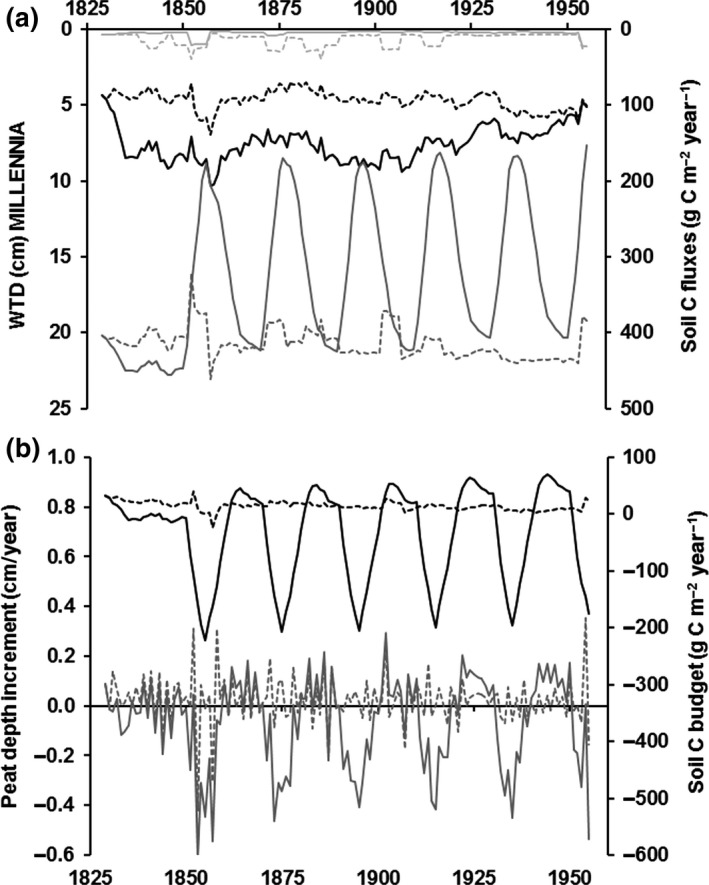
MILLENNIA model predictions (5‐year running means) of management impacts during 1825 until 1945 on (a) annual water‐table depth (WTD; black line) and carbon (C) fluxes of CO_2_ (dark gray line) and CH_4_ (light gray line), and (b) annual peat depth increments (gray line) and the soil C budget (black line) with positive numbers representing net C gains. Unbroken lines represent the unmanaged (no shoot) “natural state”, whereas dashed lines represent predictions for grouse moor management (1842–1951) including active drainage (1931–1920); drainage efficiency 25 years at maximum plus 15 years reduction to 60% and 0% by 1955) and burning (20 year cycles; with an exponential 10‐year regrow period to full net primary productivity) during 1850 till 1950

The MILLENNIA model scenario period of 1851–1950 (Figure 8) showed a positive (C gain) annual soil C budget (Figure 8b) of 12.7 ± 22.7 g C/m^2^ for the unmanaged scenario (with a mean WTD of 4.7 ± 1.5 cm; Figure 8a) compared to a negative (C loss) budget of −38.6 ± 110.1 g C/m^2^ for the grouse managed scenario with drainage (mean WTD of 7.7 ± 2.1 cm) assuming a 10‐year NPP recovery time after burning (resulting in 51.3 g C m^−2 ^year^−1^ less soil carbon gain under grouse management). The corresponding peat depth increments predicted by the MILLENNIA model during 1851–1950 showed a mean peat accumulation of 0.03 ± 0.59 cm for the unmanaged (no shoot) vs. a loss of −0.06 ± 0.61 cm for the grouse managed scenario (i.e. 0.09 cm/year less peat depth increment). Moreover, the 5‐year NPP regrowth scenario (data not shown) resulted in about 45% lower mean annual soil C budget loss (−21.6 ± 93.7 g C/m^2^) and half the peat increment loss (−0.03 ± 0.63 cm) compared to the 10‐year NPP recovery scenario. In comparison to the default burn and drain scenario, the burn but no drain 10‐year NPP regrowth scenario (data not shown) resulted in about 30% lower C and peat accumulation losses with a mean annual soil C budget of −28.4 ± 108.7 g C/m^2^ and −0.04 ± 0.63 cm mean annual peat depth increment (with a mean WTD of 4.8 ± 1.9 cm). The burn but no drain 5‐year NPP regrowth scenario reduced this further (i.e. 80% lower soil C and peat increment losses with a mean annual soil C budget of −10.9 ± 92.6 g C/m^2^ and a mean annual peat depth increment of −0.01 ± 0.64 cm.

During 1831–1850, the period of drainage only (i.e. no burning), the managed scenario reduced the mean annual soil C budget by 27.8 g C/m^2^ to −7.2 ± 15.8 g C/m^2^, which reflected an average reduction in mean annual WTD by 3.9 cm to 8.3 cm (Figure 8a). These changes in soil C budget under drainage only corresponded to an annual peat depth increment reduction by 0.06 cm to −0.02 ± 0.63 cm compared to the unmanaged scenario (Figure 8b).

The grouse moor management (i.e. 1851–1950) not only impacted C dynamics via reduced water‐tables from drainage (Figure 8a), it also altered C inputs and thus C dynamics via reduced NPP following burning. Overall, drained and 10‐year NPP recovery scenarios reduced both mean annual C losses from soil CO_2_ fluxes (308 ± 106 g C) and annual CH_4_ emissions (4.9 ± 8.3 g C) compared to the unmanaged scenario for which mean annual values were 417 ± 60 g C for CO_2_ and 13.2 ± 20.4 g C for CH_4_ net emissions (i.e. including methane oxidation, ebullition and plant‐mediated transfer (PMT) processes via sedge leaves and stems). However, whereas the no drain 10‐year NPP burn scenario decreased CO_2_ (292 ± 110 g C) and increased CH_4_ (9.8 ± 18.0 g C) emissions (Figure 8a), the 5‐year NPP scenario (data not shown) increased both CO_2_ fluxes (348 ± 92 g C) and net CH_4_ emissions (11.2 ± 18.8 g C) emissions, reflecting quicker vegetation regrowth and thus NPP and PMT recovery.

## 4 DISCUSSION

This study provided novel insights into ecological applications of using TA‐derived WTD reconstructions in a site‐specific model validation context. The findings highlight the value of combining palaeo‐ecological records with process level modeling to allow better understanding of the effects of climate and management on peat development and C cycling. This combination shows promising potential in allowing to understand the contributions of past environmental (e.g. climate) and human‐induced (e.g. grouse management) changes in peatland development over time, C stocks and corresponding C fluxes.

Although the EU transfer function predictions (as used in Amesbury et al., 2016) showed wetter conditions overall (Figure 2), the model fit to the MILLENNIA predictions was not improved, indicating a better overall fit using the geographically closer NE transfer function, possibly applying across the wider UK context, which has yet to be tested. Previous work comparing TA to recent short‐term monitored WTD by Amesbury et al. (2016) was based around a collection of samples from several sites and did not compare TA‐derived reconstructions to site‐specific long‐term modeled hydrological time series. Moreover, whereas Amesbury provided a WTD reconstruction based on comparing two different TA transfer functions, we identified and adjusted for a generic transfer function offset based on a site‐specific validation period (Figure 3) to allow a context‐dependent (i.e. NE blanket bog at Moor House) model comparison over several centuries. The fairly constant offset could well reflect a TA sample bias of summer sampling and thus drier conditions overall (i.e. during contemporary sampling to obtain TA calibration data on moisture or WTD relationships); water‐tables at Moor House frequently drop to 20–30 cm during July and August (Carroll et al., 2015). With fairly constant water‐table drawdown during drier periods (unpublished data by A. Heinemeyer et al.), a general offset can be expected (i.e. wetter sites are always wetter and vice‐versa).

Notwithstanding the overall good fit from the offset corrected TA‐derived vs. model predicted water‐tables (Figure 4), some resulting TA‐derived water‐table predictions such as the very wet period between 1730 and 1830 (as shown in Figure 6) might seem questionable. However, the predicted standing water conditions of up to −10 cm when applying the offset adjusted TA functions (using the offset corrected NE transfer function as shown in Figure 3a) fall within the tolerance and optima values (i.e. Turner et al., 2013) of two wet to aquatic TA indicator taxa (see Figure 5 in Amesbury et al., 2016), *Amphitrema wrightianum* type (2 ± 6 cm mean WTD) and *Archerella flavum* (8 ± 7 cm mean WTD). This coincides with documented wet bog conditions across Ireland (Swindles et al., 2013) and Northern Britain (Mauquoy, van Geel, Blaauw, & van der Plicht, 2002) during this period (i.e. the Little Ice Age) with potentially standing water for most of the year or water pools in hollows. Such conditions of standing water are not specifically captured by either the model or TA WTD predictions, which are relatively insensitive to water‐tables above the surface. Moreover, the climate data used in the model become less reliable before the 1850s, and this period might well have been wetter than the by Morris et al. (2015) reconstructed climate record suggests. Therefore, MILLENNIA predicted WTD during 1730–1830 might be under predicting the WTD in relation to uncertain climate input data.

Most interesting was the period between 1840s and 1940s, an unexpectedly drier period based on TA predicted hydrological conditions compared to the wetter conditions predicted by the MILLENNIA model based on climate only (Figure 6). Moor House was a formal shooting estate throughout exactly this period (i.e. 1842–1951), based on grouse bags and predator control information (ECN data provided by Rob Rose from the Centre for Ecology and Hydrology (CEH) Lancaster, personal communication). Grazing intensity on true blanket bogs such as Moor House is low (0.5 sheep/ha according to Rawes & Heal, 1978), except perhaps for recently burnt areas; historically grazing was probably low overall because farmers were unable to grow sufficient feed to maintain their stock over the winter, particularly at altitudes above 500 m. Therefore, burning in connection with drainage seems to be the most likely factor explaining the water‐table lowering. The available anecdotal evidence (R. Rose (CEH), *personal communication*) from some of the game keepers in the latter part of the period suggest that an average of 250 acres were burnt each year out of a total area of about 5,000 acres (i.e. averaging a 20 year burn cycle) and drainage ditches were maintained to aid the heather management. Such intense grouse moor management would have most likely resulted in a lowering of the water‐table by around 5 cm, as has been observed in a grip blocking study by Wilson et al. (2010). Interestingly, the MILLENNIA scenarios simulating such grouse moor management resulted in the modeled WTD capturing this drier period as inferred from the TA record, which in the model scenario was mainly a result of drainage.

Moreover, the managed model scenario indicated considerable losses in the soil C budget and decreased peat accumulation rates. Up‐scaled to an intense grouse moor management period of 100 years these accumulated C losses (i.e. 1850–1950) relate to around 5 kg C/m^2^ less soil carbon or 10 cm lower peat depth accumulation than predicted for the unmanaged scenario. Notably, burning and drainage contributed equally to the carbon loss, via reduced NPP (i.e. less litter input) and enhanced decomposition (i.e. lower WTD). Garnett et al. (2001) indicated a carbon loss and lower peat accumulation on burn management at Moor House (based on experimental burn plots) of around 73 g C m^−2^ year^−1^, slightly higher but very similar to our model predictions of 51 g C m^−2^ year^−1^. However, the Garnett et al. study contained some methodological uncertainties in relation to calculating C stocks (Clay, Worrall, & Rose, 2010) and the MILLENNIA model did not account for any potential charcoal impacts on hydrology and decomposition. In particular, impacts on peat bulk density (i.e. charcoal pieces entering peat pores) with possible changes in water holding capacity and charcoal inputs representing an inert C pool (i.e. biomass partly by‐passing decomposition; see Clay et al., 2010) with likely additional effects on decomposition rates (e.g. negative priming; see Lu et al., 2014) might need to be considered in future model developments. Notably, the Garnett et al. (2001) study has been highlighted by Evans et al. (2014) as the only substantial study assessing burning impacts on UK blanket bogs. Therefore, burn management implications on the long‐term peat C stock remain uncertain.

Although the estimated mean annual soil CO_2_ fluxes of around 300–400 g C m^−2^ year^−1^ (i.e. range of managed vs. unmanaged scenario, respectively) are within the same order of magnitude as reported for a study at Moor House (Clay et al., 2010), the CH_4_ emissions of around 5–10 g C m^−2^ year^−1^ were slightly higher compared to published values for the Flow Country (4.3 g C m^−2^ year^−1^; Levey & Gray, 2015) and Moor House (3.9 g C m^−2^ year^−1^; Worrall, Armstrong, & Adamson, 2007; and 6.4 g C m^−2^ year^−1^; Worrall, Burt, Rowson, Warburton, & Adamson, 2009). However, the existing data on net CH_4_ emissions from peatlands are very uncertain, with many older studies using inadequate methodologies (i.e. long chamber incubation periods and inaccurate analyser techniques such as gas chromatographs). Moreover, recent data from Moor House during two wet and warm years (2015–2016) measured much higher than previous average annual CH_4_ emissions from peat decomposition of 2 to 400 g C m^−2^ year^−1^ (unpublished; pers. comm. Rob Rose at CEH). However, the largest uncertainty for field measurements is likely related to manual chamber measurements often not capturing plant‐mediated transfer rates and ebullition, bypassing methane oxidation and thus leading to an overall underestimation of “true” methane fluxes. As a result of both model and measurement uncertainties, the presented CH_4_ emissions should thus not be taken as real flux predictions but rather be seen as indicators of possible differences to be assessed by future monitoring.

In conclusion, peat cores provide a valuable archive for reconstructing peat development in relation to past climate, particularly TA‐based water‐table reconstructions as a driver of peat and carbon accumulation. We have shown here that combining this peatland archive with actual site measurements and model scenarios of past water‐tables can provide additional information on likely impacts of land management, which are not easily detectable otherwise, but are crucial for explaining observed peat accumulation not explained by climate alone.
